# Sialoglycan-binding patterns of bacterial AB_5_ toxin B subunits correlate with host range and toxicity, indicating evolution independent of A subunits

**DOI:** 10.1016/j.jbc.2022.101900

**Published:** 2022-04-07

**Authors:** Naazneen Khan, Aniruddha Sasmal, Zahra Khedri, Patrick Secrest, Andrea Verhagen, Saurabh Srivastava, Nissi Varki, Xi Chen, Hai Yu, Travis Beddoe, Adrienne W. Paton, James C. Paton, Ajit Varki

**Affiliations:** 1 Glycobiology Research and Training Center, University of California San Diego, San Diego, California, USA; 2 Department of Cellular & Molecular Medicine, University of California San Diego, San Diego, California, USA; 3 Department of Chemistry, University of California Davis, Davis, California, USA; 4 Department of Biochemistry and Molecular Biology, Monash University, Clayton, Victoria, Australia; 5 Department of Animal, Plant and Soil Science and Centre for Agri Bioscience (Agri Bio), La Trobe University, Bundoora, Victoria, Australia; 6 Department of Molecular and Biomedical Science, Research Centre for Infectious Diseases, University of Adelaide, Adelaide, South Australia, Australia

**Keywords:** evolution, bacterial, toxin, phylogenetic, *Yersinia pestis*, host range, sialoglycan microarray, cytotoxicity, pathogenesis, ACK, ammonium–chloride–potassium, FACS, fluorescence-activated cell sorter, FCS, fetal calf serum, LT, heat-labile enterotoxin, MTT, 3-(4,5-dimethylthiazol-2-yl)-2,5-diphenyltetrazolium bromide, Neu5Ac, *N*-acetylneuraminic acid, Neu5Gc, *N*-glycolylneuraminic acid, PBMS, peripheral blood mononuclear cell, Plt, Pertussis-like toxin, Sia, sialic acid, Stx, Shiga toxin

## Abstract

Many pathogenic bacteria secrete AB_5_ toxins that can be virulence factors. Cytotoxic A subunits are delivered to the cytosol following B subunit binding to specific host cell surface glycans. Some B subunits are not associated with A subunits, for example, YpeB of *Yersinia pestis*, the etiologic agent of plague. Plague cannot be eradicated because of *Y. pestis*' adaptability to numerous hosts. We previously showed selective binding of other B_5_ pentamers to a sialoglycan microarray, with sialic acid (Sia) preferences corresponding to those prominently expressed by various hosts, for example, *N*-acetylneuraminic acid (Neu5Ac; prominent in humans) or *N*-glycolylneuraminic acid (Neu5Gc; prominent in ruminant mammals and rodents). Here, we report that A subunit phylogeny evolved independently of B subunits and suggest a future B subunit nomenclature based on bacterial species names. We also found *via* phylogenetic analysis of B subunits, which bind Sias, that homologous molecules show poor correlation with species phylogeny. These data indicate ongoing lateral gene transfers between species, including mixing of A and B subunits. Consistent with much broader host range of *Y. pestis*, we show that YpeB recognizes all mammalian Sia types, except for 4-*O*-acetylated ones. Notably, YpeB alone causes dose-dependent cytotoxicity, which is abolished by a mutation (Y77F) eliminating Sia recognition, suggesting that cell proliferation and death are promoted *via* lectin-like crosslinking of cell surface sialoglycoconjugates. These findings help explain the host range of *Y. pestis* and could be important for pathogenesis. Overall, our data indicate ongoing rapid evolution of both host Sias and pathogen toxin-binding properties.

Secreted bacterial AB_5_ toxins are an important class of virulence factors, named after their unique architecture, typically comprising a single catalytically active toxic A subunit and pentameric B subunits (1, 2, 3). Such AB_5_ toxins classically exert effects on host cells *via* two steps: the five B subunits form a ring-shaped pentamer responsible for binding host cell glycans and mediating uptake of holotoxin; this is followed by inhibition of host cellular functions mediated by toxic effects of the A subunit. Some of these toxins are known to recognize specific sialylated glycan moieties on target cells through a glycan-binding pocket in their B subunit (4, 5, 6, 7). A notable exception is Shiga toxin (Stx), which binds globo series of glycolipids (1).

AB_5_ toxins are typically classified and named based on the catalytic activity of their A subunits. The cholera toxin family includes Ctx and *Escherichia coli* heat-labile enterotoxins (LTs), LT-I and LT-II (8), and a less-well characterized toxin from *Campylobacter jejuni*. All these toxins have A subunits with ADP-ribosylation activities targeting an arginine of G_s_α (the α subunit of the stimulatory trimeric G protein) (1). Pertussis toxin (Ptx) produced by *Bordetella pertussis* (the causative agent of whooping cough) has a catalytic subunit with similar ADP-ribosylase activity but is placed in a separate family because in contrast to all the other AB_5_ toxins, its B subunit is heteropentameric rather than homopentameric (9). A further AB_5_ toxin variant (referred to as Plt [Pertussis-like toxin] or typhoid toxin) is produced by the typhoid fever–causing agent *Salmonella enterica* serovar Typhi. Plt has an A subunit (PltA) with ADP-ribosylase activity and a homopentameric B subunit (PltB), which collectively act as a delivery vehicle for an additional toxic subunit related to cytolethal distending toxin, resulting in a novel A_2_B_5_ architecture (6). The Stx family members, produced by *Shigella dysenteriae* and certain strains of *E. coli*, have A subunits with RNA-*N*-glycosidase activity that inhibits eukaryotic protein synthesis (1). A subset of Shiga toxigenic *E. coli* strains also produce subtilase cytotoxin (SubAB), the prototype of a fourth AB_5_ toxin family. The A subunit SubA is a subtilase family serine protease with exquisite specificity for the essential endoplasmic reticulum chaperone binding immunoglobulin protein (BiP)/glucose-regulated protein 78 (GRP78) (3, 10). An additional family is exemplified by an extraintestinal *E. coli* toxin EcxAB, the A subunit of which is a metzincin-type metalloprotease (11). Microbes that express AB_5_ toxins have a wide range of pathological effects on human populations, from travelers’ diarrhea caused by enterotoxigenic *E. coli* strains to the more serious and life-threatening diarrhea caused by *Vibrio cholerae* (12, 13), and systemic disease such as typhoid fever and the hemolytic uremic syndrome triggered by distinct *E. coli* strains producing Stx and possibly also SubAB (5, 14).

Despite sharing a similar structural architecture, the B pentamers of the various AB_5_ family members differ widely in their host cell surface receptor specificity and intracellular trafficking. For example, PltB selectively recognizes the sialic acid *N*-acetylneuraminic acid (Neu5Ac), which is very prominent in humans because of an inactivating mutation in the *CMAH* gene encoding the CMP-*N*-acetylneuraminic acid hydroxylase that converts Neu5Ac to *N*-glycolylneuraminic acid (Neu5Gc) in most other mammals (15). On the other hand, *E. coli* SubB prefers binding to Neu5Gc over Neu5Ac (5). SubB is closely related to PltB (50% identity and 68% similarity over 117 amino acids). However, SubB is even more closely related (56% identity and 79% similarity over 136 amino acids) to a putative exported protein of *Yersinia pestis* (accession no.: YPO0337, now WP_002209112) (10), although curiously, there is no associated A subunit.

*Y. pestis*, the etiologic agent of plague, was responsible for major devastating epidemics in human history that claimed millions of lives (16) and is still considered of global importance to public health and potentially in biological warfare. It was first isolated by Alexandre Yersin during the third pandemic plague that reached Hong Kong (17). Plague is predominantly a zoonosis, and a *Y. pestis* reservoir is maintained in animals such as ground squirrels and prairie dogs and transmitted to humans by fleas. *Y. pestis* also circulates in its rodent reservoir by flea-borne transmission (18). One very interesting feature of *Y. pestis* is its adaptability to more than 200 hosts (19). In addition to *Y. pestis*, the genus *Yersinia* also includes *Yersinia pseudotuberculosis* and *Yersinia enterocolitica*, which are also associated with human infections and cause mild diarrhea (20). *Y. pestis* is thought to be a “young” pathogen that diverged from the more environmental stress-tolerant and less pathogenic bacterium *Y. pseudotuberculosis* around 5000 to 7000 years ago by adapting to a flea-transmitted life cycle and the capability to cause host systemic infection (21, 22). *Y. pestis* is now considered a clonally expanded genomically degenerating variant of *Y. pseudotuberculosis*. During its evolution, *Y. pestis* showed a varied mutation rate, not strictly obeying a constant evolutionary clock (23). Three forms of plague are usually described, including bubonic, pneumonic, and septicemic plague (20). *Y. pestis* can be transmitted by flea bites causing lymph nodes to swell and form buboes (known as bubonic plague) and respiratory droplets from one person to another (causing pneumonic plague). However, when *Y. pestis* spreads *via* blood, it causes septicemic plague (20). Humans get infected either by flea bites or contact with the infected pets/domestic animals (causing conjunctivitis, skin plague, or pneumonic plague) (24). If bubonic plague is not recognized and treated in time, it can develop into pneumonic plague or systemic plague. Septicemic plague has a very high mortality rate and can also be caused directly by blood infection with the pathogen through a cut (25).

We report here for the first time that the putative *Y. pestis* B subunit binds to and is intrinsically toxic toward cells that express Neu5Ac and/or Neu5Gc. These findings are consistent with *Y. pestis* adaptability of having more than 200 hosts and provide insights into the molecular bases for its host specificity, in understanding fundamental cellular functions and potential treatment of the disease.

## Results and discussion

### A and B subunits of AB_5_ bacterial toxins evolve independently of each other

**Figure 1:**
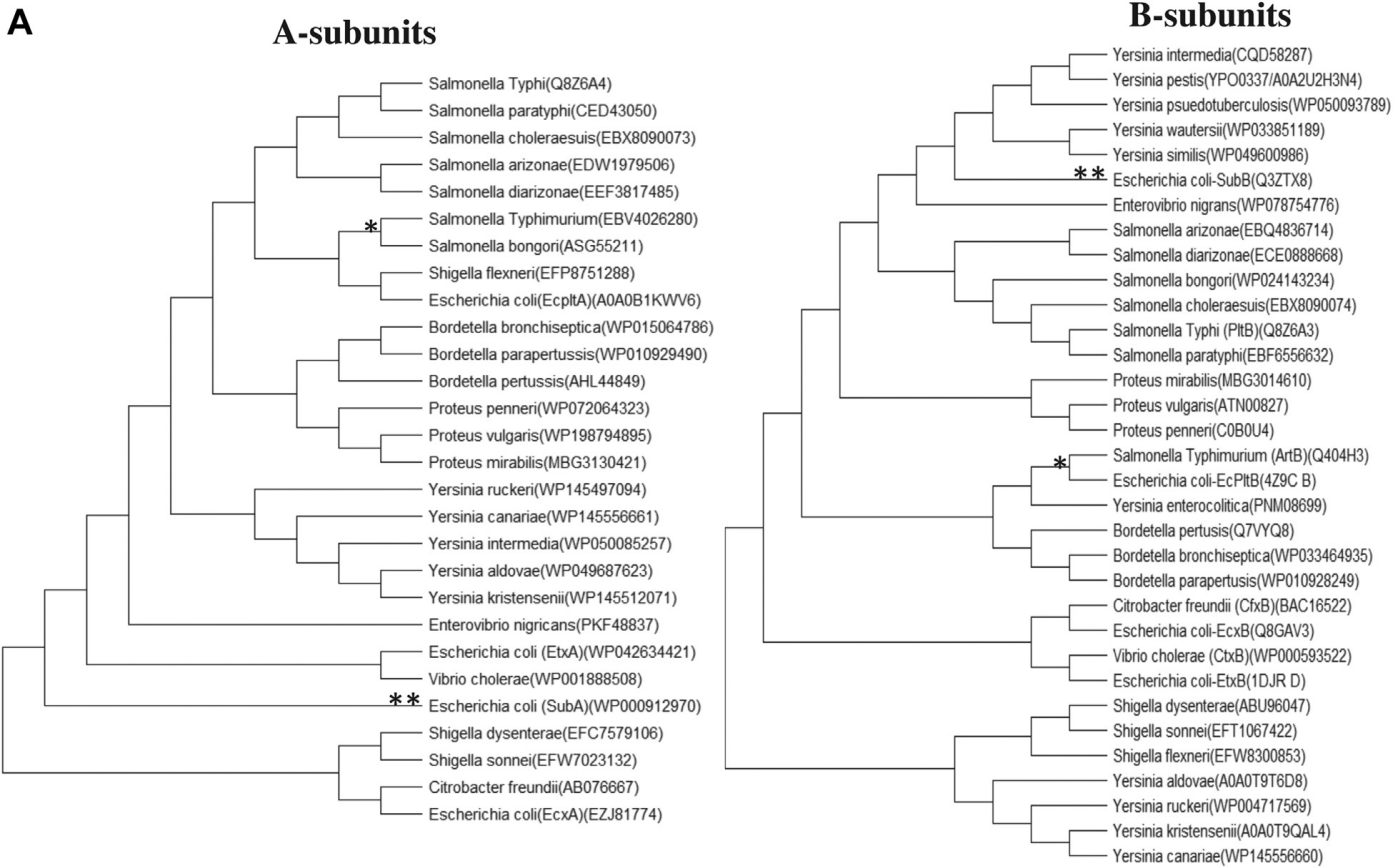

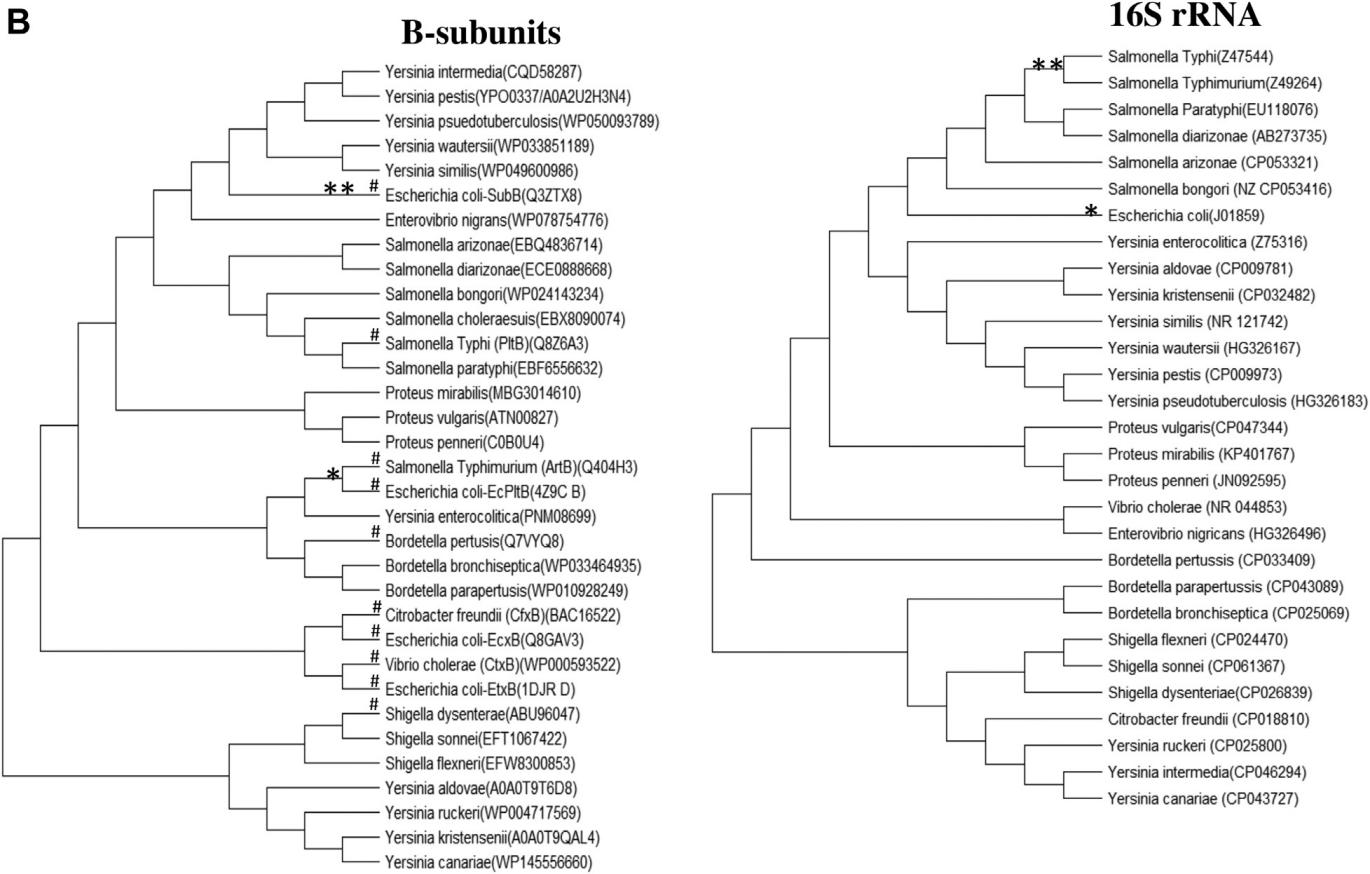
**Phylogenetic relationship of A-B subunit and B subunit-16S rRNA among different bacterial species.***A*, comparative phylogenies of A and B subunits of AB_5_ bacterial toxin show independent evolution of both subunits. ∗ shows phylogenetic relationship of *Salmonella* Typhimurium with other bacterial species with respect to A and B subunits, and ∗∗ indicates *Escherichia coli* sharing its clade with other bacterial species based on A and B subunits. (See details of the methodology in the “Experimental procedures” section.) *B*, phylogenies of AB_5_ toxin B subunit showing partial discordance with the species phylogeny based on 16S rRNA. ∗ shows *S.* Typhimurium phylogenetic relationship with other bacterial species with respect to B subunit and 16S rRNA, and ∗∗ indicates *E. coli* sharing its clade with other bacterial species based on B subunit and 16S rRNA. (See details of the methodology in the “Experimental procedures” section.) # represents published AB_5_ toxin B subunits with known host sialic acid–binding patterns.

With the development of high-throughput technologies and well-organized databases, there is a major increase in availability of bacterial AB_5_ toxin sequence data. Based on phylogenetic analysis of existing genomes, it is evident from Figure 1*A* that there is a lack of evolutionary relationship between the A and B subunits. For example, while the A subunit (ArtA) of *Salmonella* Typhimurium ArtAB shares its clade with its homolog in *S. bongori*, the B subunit (ArtB) shares a clade with a homolog from *Y. enterocolitica* and *E. coli* EcPltB (Fig. 1*A*, see also the study by Sasmal *et al.*, 2021, bioRxiv, 2021.05.28.446191). Likewise, while the *E. coli* toxin SubAB shares little similarity with other species with respect to its A subunit (SubA), it shares a clade with proteins from *Yersinia* species with respect to the B subunit (SubB). This situation has likely arisen because of lateral transmission and mixing and matching of subunit-encoding genes during the ongoing evolutionary arms race of bacterial species and toxins with respect to the host specificity. The fact that many AB_5_ toxin operons are encoded by bacteriophages and/or conjugative plasmids means that their distribution among species is also influenced by plasmid compatibility and phage host specificity.

### Phylogenies of B subunits are also partially discordant with species phylogeny

To understand the phylogeny of the various B subunits, we further compared it with species phylogeny based on 16S rRNA. It is evident from the phylogenetic tree (Fig. 1*B*) that B subunits are also evolving relatively independent of each other, in relation to species phylogeny. From the phylogenetic tree (Fig. 1*B*), there is a partial discordance even between B subunit and species phylogeny. For example, *E. coli* SubB shares a clade with homologs produced by *Yersinia* species, whereas in species phylogeny, the *E. coli* strains that produce SubB share a clade with *Salmonella* species. Similarly, *S.* Typhimurium ArtB shares its clade with homologs in *Y. enterocolitic*a but shares similarity with other *Salmonella* species based on 16S rRNA. Since the A and B subunits are independent and their biological roles are also distinct, a B subunit nomenclature based on the A subunit seems no longer logical. On the other hand, making changes in existing terminology will lead to confusion regarding the prior literature. We suggest that existing names remain unchanged going forward, but that the nomenclature of an entirely novel toxin should be related wherever practical to the bacterial species producing it (Table 1). Accordingly, the novel exported protein that appears to be an isolated *Y. pestis* B subunit is hereby named YpeB (Table 1). Having said that, the laboratory of one of the coauthors previously referred to *S.* Typhi PltB as *S.* Typhi ArtB in the literature (26) because of the close similarity of both the A and B subunits to ArtAB of *S.* Typhimurium (already described by Saitoh *et al.*, 2005 (27)) and the clear dissimilarity between the *S.* Typhi homopentameric B subunit and the heteropentameric B subunit of pertussis toxin (Plt stands for pertussis-like toxin).

**Table 1:** Current and proposed nomenclature of B5 subunit toxins

Bacterial species	Holotoxin name	A subunit name	B subunit name	Citation
*Vibrio cholerae*	Cholera toxin	CtxA	CtxB	(50)
*Salmonella enterica* serovar Typhi	Typhoid toxin	PltA	PltB, PltC	(6, 51)
*Yersinia pestis*		—	YpeB	(10); This study
*Yersinia enterocolitica*		—	YenB	This study
*Salmonella* Typhimurium		ArtA	ArtB	(7)
Enterotoxigenic *Escherichia coli*	Labile enterotoxin I Labile enterotoxin II	LT1A LT2A	LT1B LT2B	(52)
Shiga toxigenic *E. coli*	Shiga toxin 1 Shiga toxin 2 Subtilase cytotoxin	Stx1A Stx2A SubA	Stx1B Stx2B SubB	(10, 53)
Extraintestinal *E. coli*		EcxA EcPltA	EcxB EcPltB	(11, 54)

### YpeB exhibits broad specificity for sialic acid–terminated glycans

**Figure 2:**
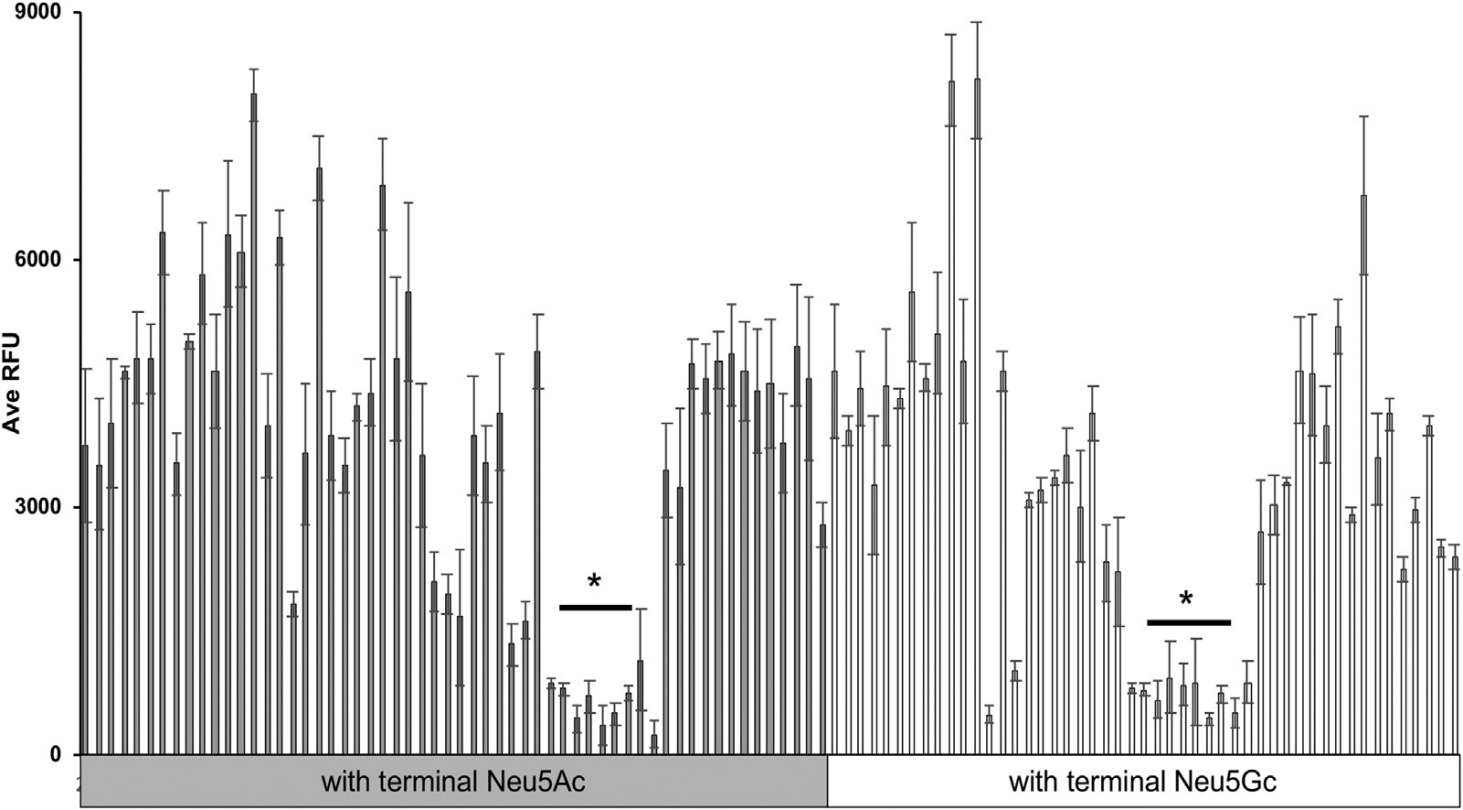
**YpeB exhibits strong binding to all Neu5Ac- and Neu5Gc-terminated glycans (except 4-*O*-acetylated, shown by *asterisk* ∗).** The *right-side bar* shows Neu5Gc-terminated glycans, and the *left-side bar* shows Neu5Ac-terminated glycans. YpeB was tested on microarray in 30 μg/ml concentration. Neu5Ac, *N*-acetylneuraminic acid; Neu5Gc, *N*-glycolylneuraminic acid.

Genomic database searches carried out as part of the original characterization of SubAB (10) identified the presence of the closely related proteins encoded by *Y. pestis* (YPO0337; 56% identity and 79% similarity over 136 amino acids) and *S.* Typhi (STY1891; 50% identity and 68% similarity over 117 amino acids), now referred to as YpeB and PltB, respectively. Interestingly, it was noted that unlike all other AB_5_ toxins known at the time, the gene encoding YpeB was not closely associated with an A subunit gene (10). This observation raises questions about its role (if any) in pathogenesis of the disease and whether it binds to host sialoglycans. PltB has been reported to bind to the human-dominant sialic acid (Neu5Ac) but not to the other major mammalian sialic acid Neu5Gc (4). Conversely, *E. coli* SubB strongly prefers to bind to the nonhuman Neu5Gc over Neu5Ac (5). In order to determine the glycan-binding patterns of YpeB, we used a customized sialoglycan array (28) to compare the ability of His_6_-tagged-YpeB and fluorescently labeled anti-His secondary antibody to bind pairs of sialylated glycans terminating in either Neu5Ac (prominently expressed in human cells) or Neu5Gc (prominently expressed in cells of most other mammals). Consistent with *Y. pestis* having >200 hosts (rats, rabbits, squirrels, chipmunks, etc), YpeB bound to both Neu5Ac and Neu5Gc terminated glycans (detailed in Fig. 2) implying a potentially broad range of host specificity. Notably, this toxin did not bind to 4-*O*-acetyl-Neu5Ac (Neu4,5Ac_2_) or 4-*O*-acetyl-Neu5Gc (Neu5Gc4Ac). The complete glycan list on the array is provided in the supporting information.

### B subunit clustering based on sialic acid–binding pattern

**Figure 3:**
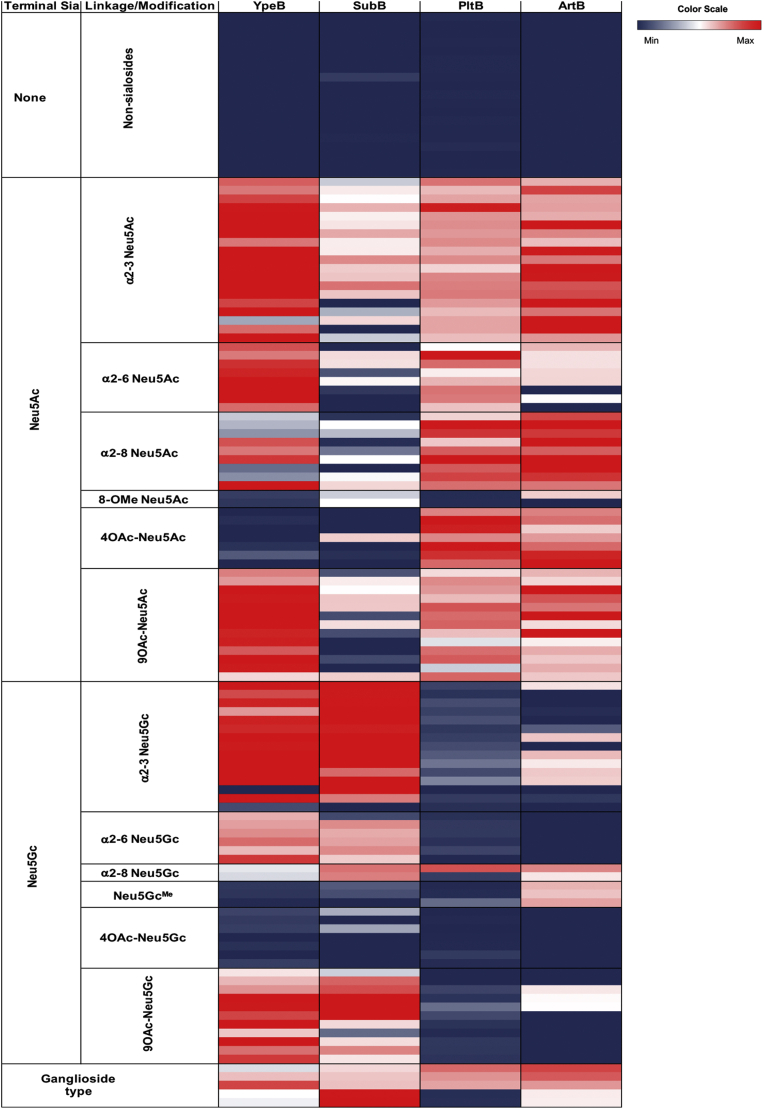
**Comparative toxin clustering of PltB, SubB, ArtB, and YpeB based on their sialic acid–binding pattern.** Average relative fluorescence units (RFUs) obtained from glycan array analysis are shown. The heatmap shows RFU value ranges from lowest (*black*) to highest (*red*). The complete glycan list is attached in the Supplementary information.

With the three already published toxins (PltB, SubB, and ArtB) having diverse sialoglycan-binding preferences and host range specificity (4, 5, 6, 7), we decided to compare the sialoglycan-binding preference of these toxins with that of YpeB (Fig. 3). We confirmed previous findings that PltB binds to the human-dominant sialic acid Neu5Ac but not to the other major mammalian sialic acid Neu5Gc, explaining a potential role for typhoid toxin in pathogenesis in humans (humans get highly symptomatic typhoid fever, but our close evolutionary relatives do not) (4). *E. coli* strains producing SubAB are commonly found in ruminants and have been associated with severe human disease (29). However, these strains also produce one or more members of the Stx family (StxAB), and so although purified SubAB is highly lethal for laboratory mammals (13), the importance of SubAB to pathogenesis of human disease is uncertain. Interestingly, however, this toxin prefers to bind to nonhuman Neu5Gc over Neu5Ac (5). The AB_5_ toxin ArtAB (27) is produced by *S.* Typhimurium, which is found in a broad range of species from cattle to humans and is a common cause of gastroenteritis. Its B subunit (ArtB) binds to both Neu5Ac and Neu5Gc (7). Interestingly, YpeB also binds to Neu5Ac and Neu5Gc and has an even broader range of host specificity. Detailed comparative sialoglycan binding of these toxins will be reported elsewhere (Sasmal *et al.*, 2021, bioRxiv, 2021.05.28.446191).

### Sialic acid–dependent binding of YpeB in Chinese hamster ovary cells

**Figure 4:**
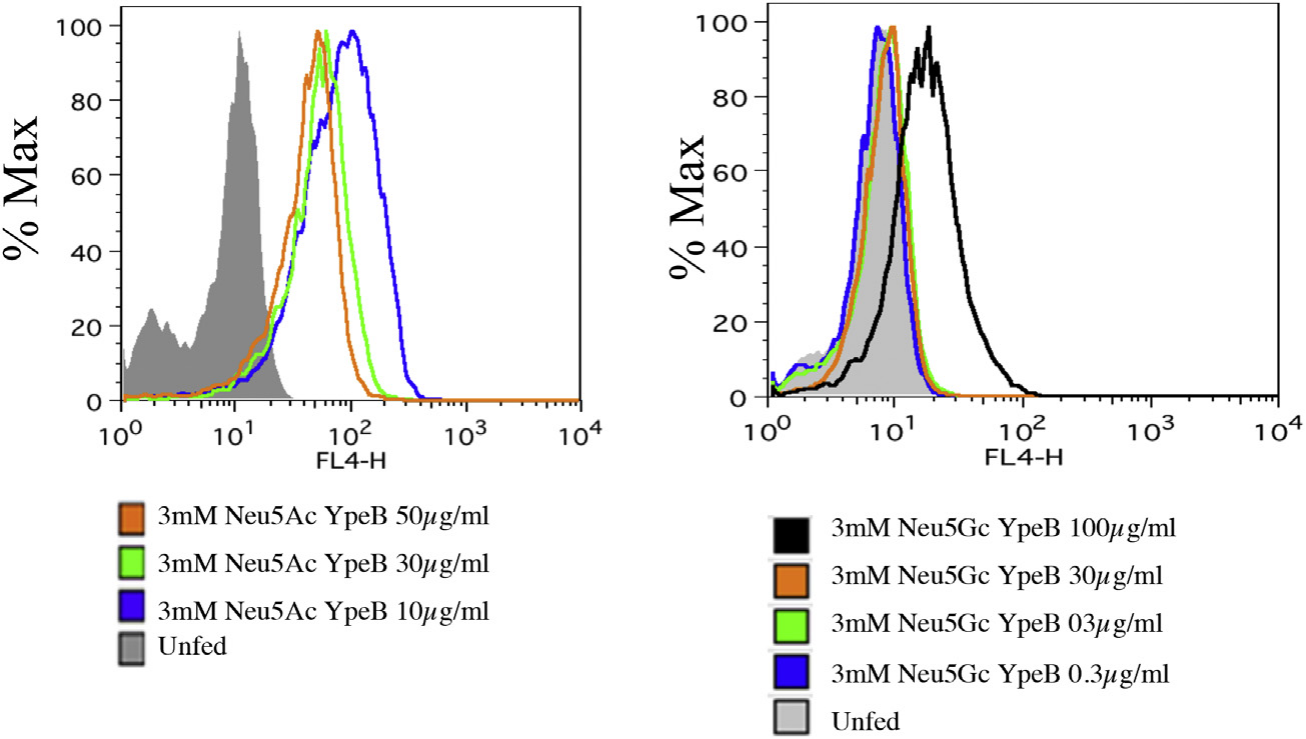
**Sialic acid–dependent binding of YpeB toxin with hyposialylated LEC29.Lec32 Chinese hamster ovary (CHO) cells fed with Neu5Ac.** The cells were cultured with 3 mM Neu5Ac for 16 h probed with different concentrations of YpeB (10, 30, and 50 μg/ml). Similarly, the cells were cultured with 3 mM Neu5Gc for 16 h probed with different concentration of YpeB (0.3, 3, 30, and 100 μg/ml), and binding was analyzed by flow cytometry. Neu5Ac, *N*-acetylneuraminic acid.

To understand if the sialoglycan-binding pattern exhibited by YpeB is Sia dependent, we used hyposialylated LEC29.Lec32 Chinese hamster ovary cells (30) and fed them with exogenous Neu5Ac or Neu5Gc and then exposed them to different concentrations of YpeB. As evident from Figure 4, the binding of YpeB to cells increases with increasing dosage of YpeB when fed with 3 mM Neu5Ac (Fig. 4). Moreover, a higher dosage of YpeB (100 μg/ml) is required to bind to Neu5Gc-fed cells as compared with Neu5Ac-fed cells (Fig. 4).

### Structural modeling and YpeB mutant impact on glycan binding

**Figure 5:**
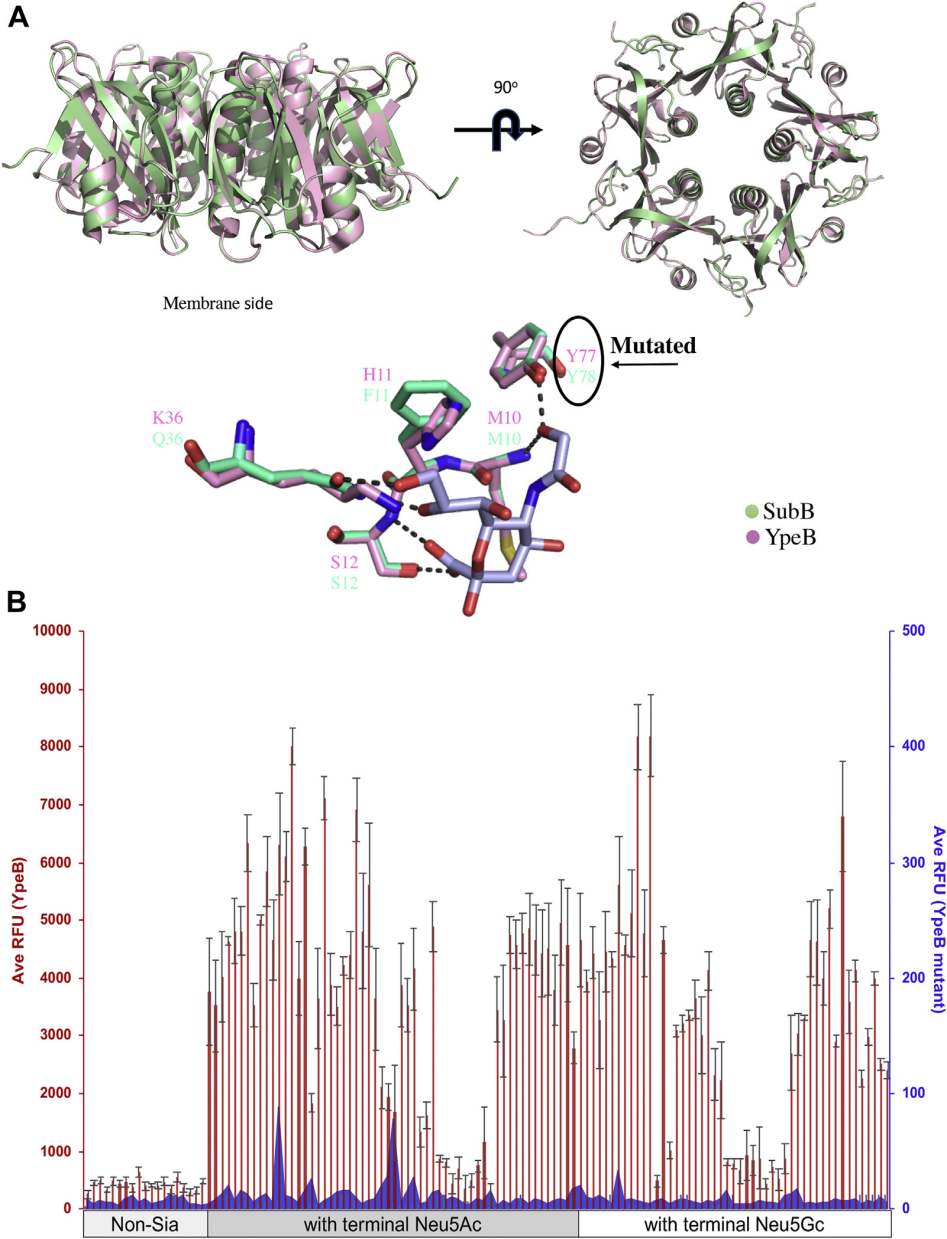
**Structural modelling and site-directed mutagenesis of YpeB.***A*, structural modeling of YpeB toxin based on *Escherichia coli* SubB (Protein Data Bank [PDB] ID: 4DWA) as a template. The sequences of *E. coli* SubB and novel YpeB are aligned, and structural modeling was done superposing SubB X-ray structure (*pale pink*) (PDB ID: 3DWP) to the model of YpeB (*pale green*) using the C-alpha coordinates of all residues. Sialic acid–binding pocket indicates Tyrosine 77 (marked as *black arrow*) to be essential for binding to host glycans. *Pink color* depicts *Yersinia pestis* YpeB, and *green color* shows *E. coli* SubB. *B*, site-directed mutagenesis (Y77F) of YpeB eliminates sialoglycan binding. The *blueshared area* showed average relative fluorescence units (RFUs) of mutant YpeB binding to sialoglycans, and *red bars* showed average RFU of YpeB binding to sialoglycans with a concentration of 30 µg/ml on glycan array.

**Figure 6:**
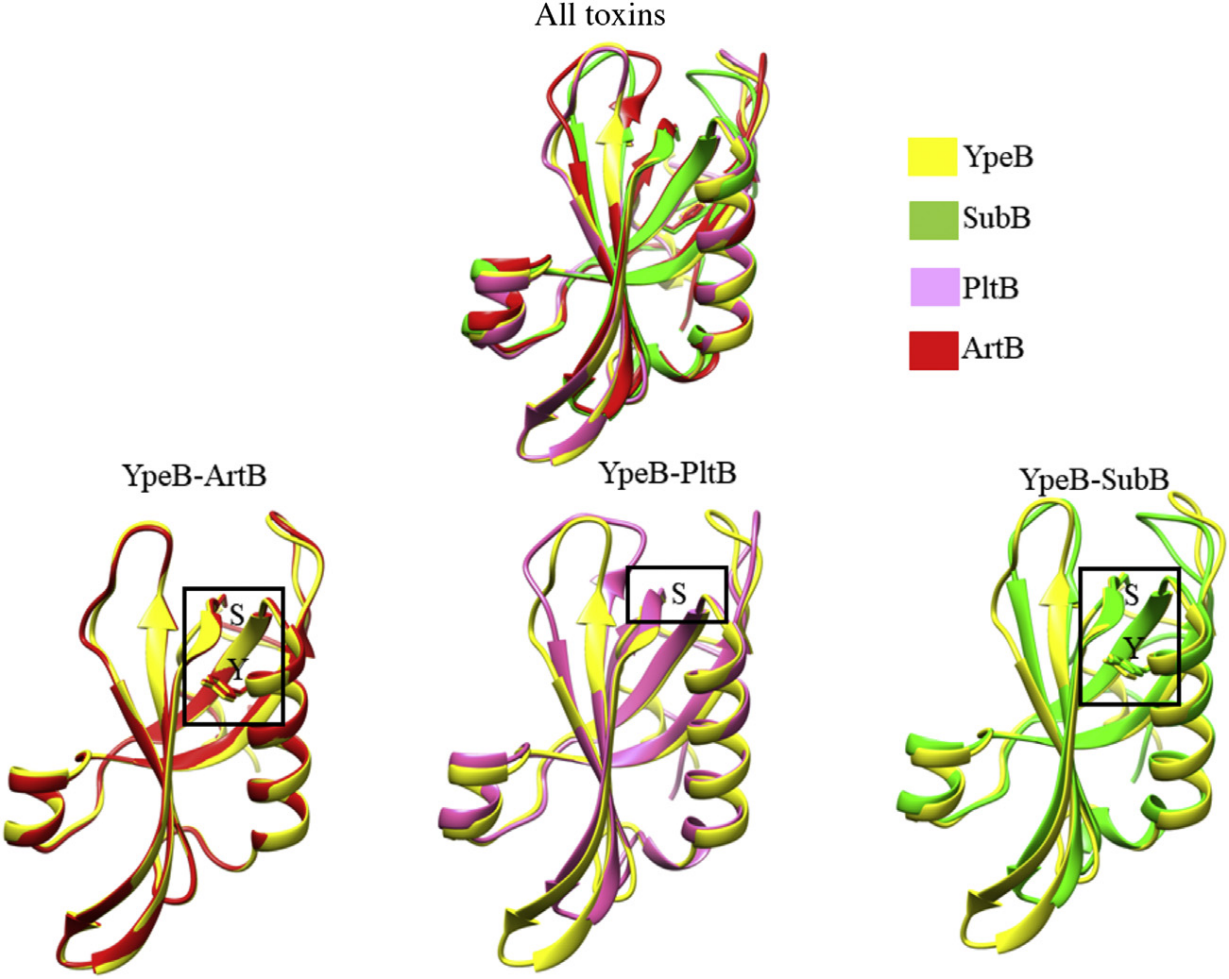
**Comparative predicted structural modeling of three toxins (PltB, SubB, and ArtB) with respect to YpeB.** The *box* represents the location of critical serine (S) and tyrosine (Y) in each toxin comparative model. Both tyrosine and serine are present in three of four toxin B subunits. Critical tyrosine (Y) is not present in *Salmonella* Typhi PltB.

Since the amino acid sequence of YpeB shares 56% identity and 79% similarity with SubB, we predicted the structure feature and amino acids of YpeB that are crucial for binding to host glycans using SubB as a template in UCSF Chimera software (Resource for Biocomputing, Visualization, and Informatics, UCSF) (Fig. 5*A*). It is well documented that two amino acids (S12 and Y78) are crucial for the binding of glycans to SubB, with a S12A substitution abolishing glycan binding completely, whereas a Y78F mutation selectively abolished binding to Neu5Gc (5). Taking advantage of structural modeling, we predicted that Y77 in YpeB might also be critical for binding to host glycans (Fig. S1) Indeed, when we mutated the amino acid residue in this position from tyrosine (Y) to phenylalanine (F) (Y77F), binding of glycans was abolished, explaining the essential role of Y77 in binding to host cell surface glycans (Fig. 5*B*). Comparative structural modeling of published toxin B subunits (PltB, SubB, ArtB, and YpeB) reported in Figure 3 is shown in Figure 6. It is clear from Figure 6 that PltB lacks an essential tyrosine that could be the reason for its selective binding to human dominant Neu5Ac. Unlike PltB, SubB contains both essential serine and tyrosine residues in its glycan-binding pocket, with the latter required for its capacity to preferentially bind Neu5Gc over Neu5Ac (5). Similarly, the ArtB structural model shows both serine and tyrosine in the glycan-binding pocket and in addition also contains an extra loop that could contribute to its broad glycan target specificity.

### YpeB induces apoptosis or necrosis in different cell lines, peripheral blood mononuclear cells, and splenocytes

**Figure 7:**
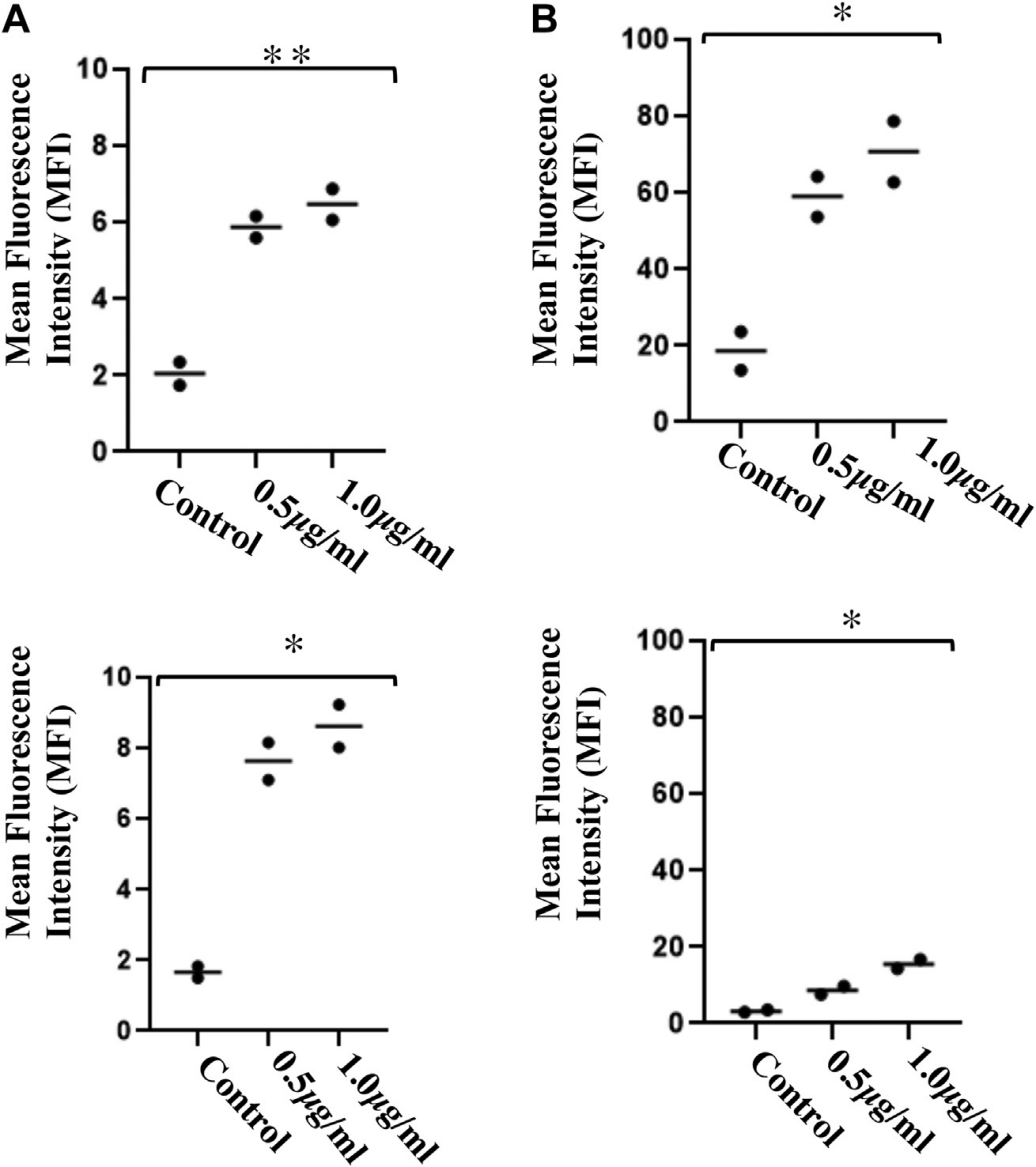
**Toxicity of YpeB on cell lines, PBMCs, and spleenocytes.***A*, Effect of YpeB on cell lines. COS-7 cells (1 × 10^6^ cells) were incubated in Dulbecco's modified Eagle's medium with YpeB (0.5 and 1.0 μg/ml) for 16 h, then labeled with propidium iodide (PI), and analyzed by flow cytometry (*upper panel*). U-937 cells (1 × 10^6^ cells) were incubated in RPMI medium with YpeB (0.5 and 1.0 μg/ml) for 16 h, then labeled with PI, and analyzed by flow cytometry (*lower panel*). The data are represented as mean fluorescence intensity (MFI) for each cell line. ∗∗*p* < 0.01, ∗*p* < 0.05. *B*, effect of YpeB on fresh mouse splenocytes and human peripheral blood mononuclear cells (PBMCs). Cells (*upper panel* mouse spleenocytes and *lower panel* PBMCs) were incubated with YpeB (0.5 and 1.0 μg/ml) for 4 h and labeled with PI and analyzed by flow cytometry. The data are represented as MFI for splenocytes and PBMC. ∗∗*p* < 0.01, ∗*p* < 0.05. (Since primary cells are more sensitive, we have reduced incubation time to 4 h).

In typical AB_5_ bacterial toxins, the B subunits mediate glycan binding, and the cognate A subunit then mediates toxicity. If so, the question arises as to why *Y. pestis* has an evolutionarily conserved isolated B subunit (A subunit is lacking), and what the role of this “orphan” B subunit might be. To ask whether the YpeB subunit without its counterpart can cause toxicity, general cytotoxicity was assessed by determining rates of necrosis in U937 human leukemia cells and COS-7 cells (from green monkey), exposed to 0.5 or 1.0 μg/ml YpeB for 16 h (Fig. 7*A*). Human peripheral blood mononuclear cells (PBMCs) and splenocytes from WT mice were also incubated with different concentrations of YpeB (1–20 μg/ml) for 4 h (Fig. 7*B*). Since PBMCs and mouse splenocytes are primary cell lines, we decreased the incubation time as well as using higher YpeB concentrations than for the cell lines. U-937 and COS-7 cell lines showed increased toxicity with increasing dose of YpeB. Similarly, PBMC and splenocytes showed increased toxicity with increased YpeB concentration. It is likely that the previously reported toxic/inflammatory effects of PltB of typhoid toxin (referred to as ArtB) on various cell types (26, 31) have an analogous mechanistic basis. Notably, the cytotoxicity of U-937 and COS-7 cell lines was not observed with the inactive YpeB toxin B subunit (Fig. S2). All the experiments related to mice were approved by Institutional Animal Care and Use Committee, University of California San Diego Board.

### Mechanism of YpeB toxicity likely involves lectin-like crosslinking of glycoconjugates

**Figure 8:**
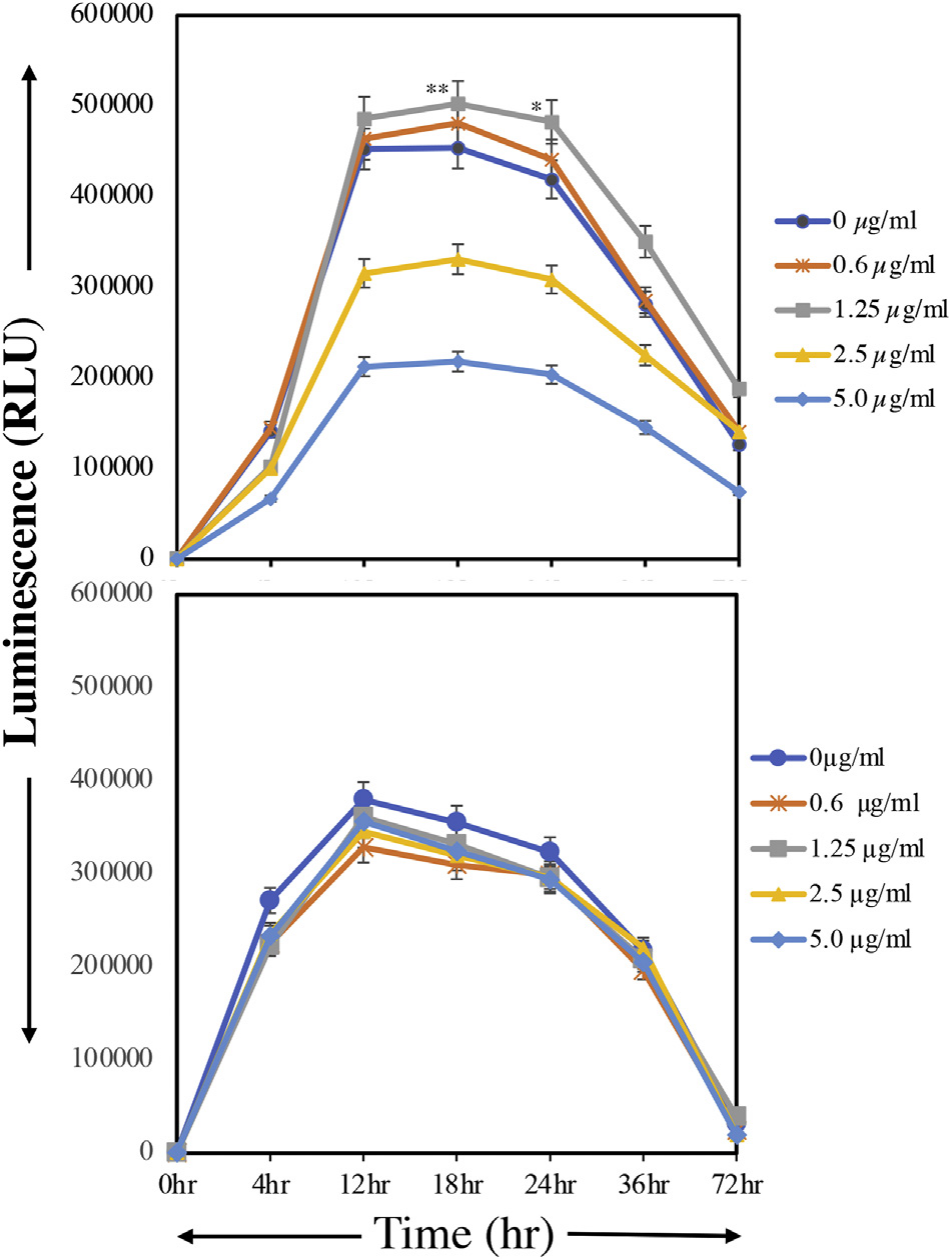
**Cell growth and viability of CHO-K1 cells (5000 cells/well) with active (*upper panel*) or nonbinding (*lower panel*) YpeB.** Cells were incubated with toxin concentrations ranging from 0.6 to 5.0 μg/ml at different time points ranging from 0 to 72 h using MTT cell viability assay on ELISA plate reader at 570 nm (described in the Experimental procedures section). The *upper panel* shows lectin-like stimulation, whereas no stimulation and proliferation were observed in *lower panel*, where cells incubated with mutant YpeB (serum-free condition and toxin added after that). ∗∗*p* < 0.01, ∗*p* < 0.05. MTT, (3-(4,5-dimethylthiazol-2-yl)-2,5-diphenyltetrazolium bromide).

CHO-K1 cells were treated with active or inactive YpeB, followed by measurement of cell viability using an MTT (3-(4,5-dimethylthiazol-2-yl)-2,5-diphenyltetrazolium bromide) assay over the next 72 h. At low concentrations of YpeB (0.6 μg/ml, 1.25 μg/ml), the toxin activates cells to proliferate, whereas, at higher concentrations (2.5 μg/ml, 5.0 μg/ml), the cells were killed (Fig. 8*A*), likely reflecting a classic lectin-like crosslinking of glycoconjugates. After reaching saturation, the cells died because they were grown in very low fetal bovine serum in the culture media. A similar scenario was observed when we incubated cells with MAL-1 lectin (Fig. S3). This lectin-like activation at low concentrations and killing at high concentrations were not observed with inactive YpeB toxin B subunit (Fig. 8*B*).

### Subcutaneous injection of YpeB toxin causes proliferation of B cells

**Figure 9:**
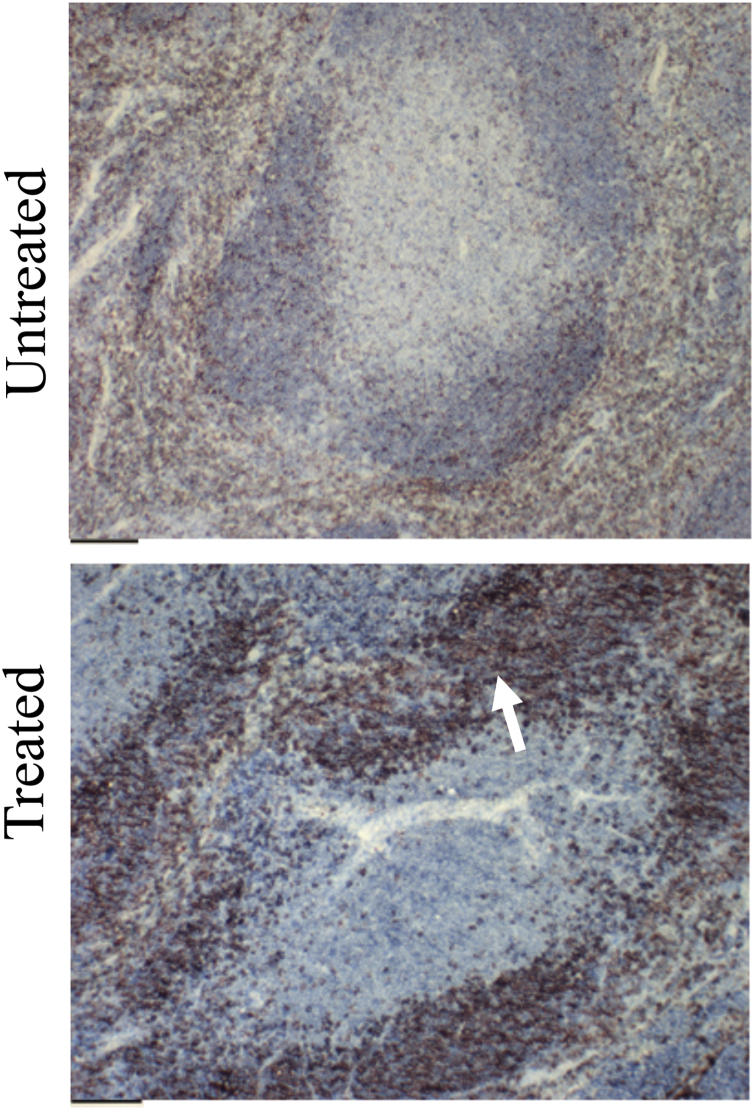
**Subcutaneous injection of YpeB causes proliferation of B cells in spleen sections.** The *upper panel* is the spleen section untreated with YpeB, and *lower panel* shows YpeB-treated spleen section. *Dark brown dots* in YpeB-treated spleen section show proliferation of B cells (stained with B220). The scale bar represents 100 μm.

Several studies have reported inflammatory responses initiated by the host immune system in response to *Y. pestis*. Among them is the hyperproliferation of B cells (32). When YpeB was injected subcutaneously, examination of spleen sections showed proliferation of B cells marked with B220 (B cell marker) (Fig. 9). This is also likely because of the lectin-like properties of YpeB.

## Conclusions and perspectives

Host specificity of pathogens is the result of various factors including genetic diversity, physiological conditions, and ecological opportunities. There are two general categories of bacterial pathogens: one group known as specialists that establish intimate relationships with single (or a closely related group of) hosts, whereas the other group known as generalists are capable of infecting a wide range of hosts. Most pathogens exhibit some degree of host preference; for example, in malaria, *Plasmodium falciparum* binds to human-dominant Neu5Ac and is specific for humans, whereas *Plasmodium reichenowi* specifically infects chimpanzees (33, 34).

The features that govern host specificity are not yet fully understood. In the present study, we have explored the evolutionary relationship between A and B subunits of bacterial AB_5_ toxins. Because of the lack of evolutionary relationship and independent evolution of these two subunits, it is evident that B subunits are evolving based on cross-species transmission between bacteria that infect a wide range of hosts.

In the present study, we have focused attention on *Y. pestis* YpeB, which is clearly a member of the AB_5_ toxin B subunit family. Among 11 species of *Yersinia*, only three are pathogenic. Among them, the deadliest is *Y. pestis*, the causative agent of plague, which was responsible for the black death of Europe in the 14th century (35). YpeB shares strongest sequence similarity and phylogenetic relationships with *E. coli* SubB and *S.* Typhi PltB. Unlike SubB and PltB, YpeB binds to both Neu5Ac- and Neu5Gc-terminated glycans as previously discovered for *S.* Typhimurium ArtB (7). The broad glycan-binding pattern of YpeB is consistent with a broad range of host specificity of a pathogen that can infect a wide variety of animals ranging from mouse, rats, rabbits, cats, dogs, and squirrels to humans (36). In terms of severity of diseases, *S.* Typhimurium causes mild gastrointestinal infections in humans (37), whereas *Y. pestis* has been involved in three human plague pandemics (38).

A comparison of YpeB and other toxin B subunits (PltB, SubB, and ArtB) glycan-binding patterns reveals differences in binding to host glycans reflecting the host range, virulence, and pathogenesis of the bacteria that produce them. PltB exhibits exquisite preference for human-specific Neu5Ac-terminated sialoglycans on surface glycoproteins that serve as its receptors (4). In contrast, SubB prefers to bind to Neu5Gc-terminated sialoglycans on surface glycoproteins that serve as its receptors (5). It is known that purified SubAB is highly lethal for mice when injected intraperitoneally, causing pathology similar to that seen in human cases of hemolytic uremic syndrome (14). This is consistent with preferential expression of Neu5Gc by rodents. Strains of *E. coli* producing SubAB have also been associated with severe disease in humans, including hemolytic uremic syndrome (10, 29). However, these strains also produce Stx (StxAB), so the contribution of SubAB to human disease/pathology remains uncertain. The binding preference of ArtB for both Neu5Ac- and Neu5Gc-terminated glycans is entirely consistent with the broad host range of the bacterium that harbors it. Interestingly, despite having broad glycan-binding patterns, YpeB lacks binding with 4-*O*-acetylated Neu5Ac and Neu5Gc glycans. Considering high expression of 4-*O*-acetylated glycans in horses (39), it is interesting that they are anecdotally noted to be less affected in plague epidemics (see also Sasmal *et al.*, 2021, bioRxiv, 2021.05.28.446191), where we describe another orphan B subunit toxin from *Yersinia* species (*Y. enterocolitica* [YenB]) with an even broader range of Sia recognition than YpeB of *Y. pestis*, again correlating with a broader range of hosts.

Unlike other AB_5_ toxin members, another interesting feature about YpeB is that, without an enzymatically active A subunit, the B subunit alone causes toxicity to different cell types. The cytotoxic effect of YpeB is likely based on binding to the target cells and a lectin-like action of the protein (40), as the Y77F glycan-binding site of mutant YpeB has no toxic effects. Toxicity was highest in mouse splenocytes as compared with human cells (cell lines and PBMCs) and monkey kidney cell lines. The higher toxicity in mouse splenocytes could be due to higher concentration of Neu5Ac- and Neu5Gc-terminating glycans (41) as compared with green monkey kidney cells. In contrast, human cells showed toxicity owing to their dominant Neu5Ac.

With the help of the already known structure of SubB, structural modeling of YpeB reveals conserved amino acids essential for host glycan binding. For *E. coli* SubB, it is evident that Ser12 and Tyr78 are essential for binding to sialoglycans. In SubB, Tyr78 interacts with the extra OH group of Neu5Gc and is thus critical for binding to Neu5Gc but does not impact the weaker recognition of Neu5Ac (5). Interestingly, the analogous Y77F mutation in YpeB abolished the binding to both Neu5Ac and Neu5Gc glycans, which indicates that the binding tyrosine in YpeB plays a more prominent role in glycan binding than it does in SubB.

Since we do not know how the B subunit alone causes toxicity without an enzymatically active A subunit, we speculated that multivalent YpeB behaves like a lectin that activates the target cell by crosslinking cell surface glycans, thereby exerting multifaceted effects such as signal modulation, membrane reshuffling, cell–cell adhesions, and thus contributes to the pathogenic property of the bacterium (42). Significantly, the nonsialic acid–binding mutant of YpeB Y77F showed reduced toxicity and cell proliferation. We have shown that YpeB exerts toxicity only at relatively high concentrations. A similar case was observed for wheat germ agglutinin, high concentrations of which kill cells (43). In addition, higher doses of *S.* Typhi PltB causes inflammation and vacuolation with less incubation time (31).

Taken together, our study is the first to report the broad range of glycan-binding specificity of YpeB that binds to both Neu5Ac- and Neu5Gc-terminated glycans (except 4-OAc-Sia glycans), which is concordant with the broad host range of *Y. pestis*. The orphan B subunit causes toxicity in different cell types of human, mouse, and monkey origin and behaves like a lectin to induce toxicity in cells. Nevertheless, more work is required to understand why YpeB did not bind to 4-OAc-Sia glycans, which are highly expressed in horses, that are speculated to be resistant to plague.

## Experimental procedures

### Phylogenetic inference of AB5 toxins among species

The genome sequence of 33 bacterial species was retrieved *via* BLAST from the National Center for Biotechnology Information (www.ncbi.nlm.nih) and UniProt, using as queries A and B subunits of the AB_5_ family toxins from *S.* Typhi (PltAB) and *E. coli* (SubAB). Sequence alignments were performed using ClustalW implemented in MEGA, version 7.0 (Pennsylvania State University) (44). Based on sequence retrieved from 33 species, a neighbor-joining phylogenetic tree was constructed. All ambiguous positions were removed for each sequence pair (pairwise deletion option). The sequence of A subunits was retrieved following the aforementioned method. Since it is known that some bacterial species lack an active A subunit, a phylogenetic tree is constructed based on the bacterial species that contain an A subunit. In addition, sequences of 16S rRNA from each species were retrieved and neighbor-joining phylogenetic tree was constructed using MEGA, version 7.0 (44). Accession numbers of the retrieved sequences were added on the phylogenetic tree.

### Purification of YpeB

The *Y. pestis ypeB* ORF was chemically synthesized (GenScript) incorporating flanking 5′ and 3′ EcoRI and HindIII restriction sites, as well as a region encoding a His_6_ tag on the 3′ terminus of *YpeB*, and was cloned into pBAD18, so that the ORF is under the control of the vector *ara* promoter. The construct was then transformed into an *E. coli* BL21(DE3) *lpxM* mutant that produces a penta-acylated lipopolysaccharide that has very low endotoxic activity. The recombinant bacterium was grown in 500 ml LB at 37 °C to late logarithmic phase, diluted 50:50 with fresh medium supplemented with 0.2% (w/v) arabinose to induce *YpeB* expression, and then incubated overnight at 26 °C. Cells were harvested by centrifugation and resuspended in 20 ml loading buffer (50 mM sodium phosphate, 300 mM NaCl, and pH 8.0) and lysed with a sonicator for 10 min (On/Off). Cell debris was removed by centrifugation at 40,000*g* for 30 min at 4 °C. The supernatant was then loaded onto a pre-equilibrated 2 ml nickel– nitrilotriacetic acid column. The nickel–nitrilotriacetic acid column was washed with 40 ml loading buffer (mentioned previously), and bound proteins were eluted with a 30 ml gradient of 0 to 500 mM imidazole in loading buffer. Ten tubes of 3 ml fractions were collected and analyzed by SDS-PAGE, followed by staining with Coomassie blue or Western blotting with monoclonal anti-His_6_ (GenScript). YpeB migrated as a single ∼16 KDa band on SDS-PAGE when stained with Coomassie blue.

### Sialoglycan microarray

The sialoglycan microarray method was adapted and modified from the literature reported earlier (45, 46). Defined sialosides with amine linker were chemoenzymatically synthesized and then quantitated utilizing an improved 1,2-diamino-4,5-methylenedioxybenzene-HPLC method. About 100 μM of sialoglycan solution (in 300 mM sodium phosphate buffer, pH 8.4) was printed in quadruplets on *N*-hydroxysuccinimide-functionalized glass slides (PolyAn 3D-*N*-hydroxysuccinimide; catalog no.: PO-10400401) using an ArrayIt SpotBot Extreme instrument (Arrayit). The slides were blocked (0.05 M ethanolamine solution in 0.1 M Tris–HCl, pH 9.0), washed with warm Milli-Q water, and dried. The slides were then fitted in a multiwell microarray hybridization cassette (ArrayIt) to divide into eight wells and rehydrated with 400 μl of ovalbumin (1% w/v, PBS) for 1 h in a humid chamber with gentle shaking. After that, the blocking solution was discarded and a 400 μl of solution of the toxin B subunit (30 μg/ml) in the same blocking buffer was added to the individual well. The slides were incubated for 2 h at room temperature with gentle shaking, and the slides were washed with PBS-Tween (0.1% v/v). The wells were then treated with Cy3-conjugated anti-His (Rockland Antibodies & Assays; catalog no.: 200-304-382) secondary antibody at 1:500 dilution in PBS. Followed by gentle shaking for 1 h in the dark and humid chamber. The slides were then washed, dried, and scanned with a GenePix 4000B scanner (Molecular Devices Corp) at wavelength 532 nm. Data analysis was performed using the GenePix Pro 7.3 software (Molecular Devices Corp). The raw data analysis and sorting using the numerical codes were performed on Microsoft Excel. Local background subtraction was performed, and data were plotted separately for each subarray. The binding specificity to glycoconjugates for each protein was plotted based on the average relative fluorescence units *versus* glycan IDs.

### Cell culture and monosaccharide supplementation

In order to determine the sialic acid–dependent binding of YpeB toxin B subunit, we used the cultured cell line LEC29.Lec32. These Chinese hamster ovary cells (that lack sialic acid production because of mutation in CMAS gene (30)). LEC29.Lec32 cells was propagated as an adherent cell line supplemented with alpha minimum essential medium with 10% (v/v) heat-inactivated fetal calf serum, 2 mM glutamine, 100 U of penicillin per ml, and 100 μg of streptomycin per ml in a humidified 5% CO_2_, 37 °C atmosphere. For medium supplementation, Neu5Ac (3 mM) and Neu5Gc (3 mM) were dissolved in PBS, titrated to a neutral pH, and filter sterilized. The sugars were added at the indicated concentrations. For the sugar turnover experiments, the cells were fed as mentioned previously, and on day 0, the cell culture medium was switched to respective medium, supplemented with 1% (v/v) Nutridoma (Roche), 100 U of penicillin per ml, and 100 μg of streptomycin without Neu5Ac and Neu5Gc.

### Flow cytometry analysis

Cells were washed with PBS and incubated with different concentrations of YpeB in PBS and incubated with Alexa Fluor 647 anti-His antibody for 30 min on ice. After an additional washing step, the cells were analyzed by flow cytometry.

### Cell culture and toxin treatment

To understand the toxicity of B subunit in cells, we cultured different cell lines: COS-7 cells (African green monkey kidney fibroblast), grown in Dulbecco's modified Eagle's medium supplemented with 10% fetal calf serum (FCS) and 50 IU of penicillin/50 μg/ml of streptomycin and U937 (human monocytes) cells, grown in RPMI1640 medium supplemented with 10 mM Hepes, 2 mM l-glutamine, 1 mM sodium pyruvate, 10% (v/v) heat-inactivated FCS, 50 IU of penicillin/50 mg/ml streptomycin, at 37 °C and 5% CO_2_. For the toxin treatment, cells were seeded into 24-well tissue culture plates (1 × 10^6^/well) and were exposed to YpeB toxin B subunit at the indicated concentration in 300 μl of culture medium. In addition, PBMC and mouse splenocytes were also cultured in RPMI1640 medium supplemented with 10 mM Hepes, 2 mM l-glutamine, 1 mM sodium pyruvate, 10% (v/v) heat-inactivated FCS, 50 IU of penicillin/50 mg/ml streptomycin, and treated with indicated concentration of toxin at different time interval and visualized under FACSCalibur (BD FACSCalibur).

### PBMC isolation and splenocytes from WT mice

Freshly drawn venous blood was used to isolate PBMC with Ficoll hypaque method. After isolating PBMCs, cells were washed with PBS and centrifuged for 10 min at 200*g*. After washing, the supernatant was discarded, and the cells were treated with ammonium–chloride–potassium (ACK) lysis buffer and incubated for 10 min. This step was repeated till the trace of red blood cells vanished. The cells were counted with hemocytometer, and approximately ∼2 × 10^6^ cells were seeded in RPMI + 10% (v/v) FCS in a tissue culture plate and incubated with toxins at different concentration. The similar steps were followed for splenocytes. The freshly isolated spleens were crushed using plunger end of the syringe and filtered through the cell strainer into the Petri dish. The single-cell suspension was centrifuged and washed three times for 10 min at 200*g*. The cells were treated with ACK lysis buffer and incubated for 10 min. After ACK lysis, the cells were suspended in RPMI + 10% FCS and seeded in a cell culture plate and incubated with 1 and 10 μg/ml toxin concentration.

### Examination of apoptosis and necrosis

Exploring the role of B subunit in toxicity, cultured cells were stained with markers of apoptosis as well as necrosis. Cells were detected by differential staining with annexin V (which stains apoptotic and necrotic cells) and propidium iodide (which stains necrotic cells only) using an Annexin-V Apoptosis Kit 2 (Invitrogen; catalog no.: V13241) according to the manufacturer's instructions. Briefly, 10^6^ cells were treated for 16 h with 10 μg/ml YpeB and transferred from the wells of the tissue culture plate into fluorescence-activated cell sorter (FACS) tubes (Falcon; catalog no.: 352008). The cells were washed three times with 3 ml annexin V-binding buffer and resuspended in 50 μl of annexin V working reagent, consisting of 1/25 annexin V–Alexa 488 (which emits at 519 nm) and 1 μg/ml propidium iodide (which emits at 617 nm) in annexin V-binding buffer. The cells were incubated at room temperature in the dark for 30 min and then washed and analyzed immediately on an FACSCalibur flow cytometer.

### Structural modeling

Since YpeB toxin B subunit is a novel toxin without a counterpart A subunit, the crystal structure through X-ray crystallography is a challenge. The structure of the YpeB was predicted using PyMOL software (Schrödinger, Inc). The highest scored structure was selected for the structural comparisons; in this case, we selected *E. coli* SubB subunit (Protein Data Bank ID: 3DWP) because of its high similarity. The sialic acid–binding site was predicted after docking it with Neu5Gc. In addition, comparison of structures of ArtB, PltB, and SubB with YpeB were done using UCSF Chimera software (47).

The YpeB amino acid sequences were aligned using the program EXPRESSO (Centre for Genomic Regulation) (48), and homology modeling was performed employing MODELLER software (University of California San Francisco) (49), using the crystal structure of SubB (Protein Data Bank ID: 3DWP) as a template. The protein sequences of the novel YpeB and mutant YpeB B subunit are provided in the supporting information (Fig. S4).

### Cell viability assay

Cell viability assays were performed in CHO-K1 cell lines. Promega MTT, absorbed into the cell and eventually the mitochondria, is broken down into formazan by mitochondria succinate dehydrogenase. Accumulation of formazan reflects the activity of mitochondria directly and cell viability indirectly. Cell viability was measured by the MTT assay. CHO-K1 cells were seeded on 96-well plates at a density of 5 × 10^3^ cells/well and cultured overnight in advanced media with 2% fetal bovine serum. Different concentrations of YpeB active and inactive toxin B subunits were added to the wells and incubated for 4 h. In addition, the plant lectin MAL-1 was also added because it lacks the active component (like YpeB) and acts as a control. After incubation, MTT was added at a concentration of 0.5 mg/ml after medium (200 μl) was added to each well. The plates were incubated at 37 °C for different time points (0, 4, 12, 24, and 72 h) and measured on an ELISA reader (Bio-Rad) at a wavelength of 570 nm, and absorbance was recorded.

### Histopathology

To understand the role of YpeB in toxicity and B-cell proliferation, *in vivo*, we injected YpeB subcutaneously into flanks of WT mice, which were euthanized and sacrificed after 3 days. Spleens were harvested and frozen for frozen sections and immunostained using several markers including marker B220 for B cells.

### Statistical analysis

All data were analyzed with GraphPad Prism, version 6.0 (GraphPad Software, Inc), and comparisons were made by Student’s *t* test (two-tailed), one-way analysis of variance, or two-way analysis of variance, as appropriate. A probability of *p* < 0.05 indicated as a statistical significance.

## Data availability

All data are contained within the article and supporting information.

## Supporting information

This article contains supporting information.

## Conflict of interest

The authors declare that they have no conflicts of interest with the contents of this article.
