# Supplementary material for: Sialoglycan-binding patterns of bacterial AB5 toxin B subunits correlate with host range and toxicity, indicating evolution independent of A subunits
Source: J Biol Chem. 2022 Apr 7;298(5):101900. doi: 10.1016/j.jbc.2022.101900 (PMC9120245; doi:10.1016/j.jbc.2022.101900)
Supplement: Supporting information [file mmc1.docx]

**SUPPORTING INFORMATION**

**Sialoglycan binding patterns of bacterial AB_5_ toxin B subunits correlate with host range and toxicity, indicating evolution independent of A subunits**

Naazneen Khan^1,2^, Aniruddha Sasmal^1,2^, Zahra Khedri^1,2^, Patrick Secrest^1,2^, Andrea Verhagen^1,2^, Saurabh Srivastava^1,2^, Nissi Varki^1,2^, Xi Chen^3^, Hai Yu^3^,

Travis Beddoe^4,5^, Adrienne W. Paton^6^, James C. Paton^6^, Ajit Varki^1,2^

^1^ *Glycobiology Research and Training Center, ^2^ Department of Cellular & Molecular Medicine, University of California San Diego, San Diego, California, USA ^3^ Department of Chemistry, University of California Davis, Davis, California, USA.^4^Department of Biochemistry and Molecular Biology, Monash University, Clayton, VIC, 3800, Australia.^5^Department of Animal, Plant and Soil Science and Centre for Agri Bioscience (Agri Bio), La Trobe University, Bundoora, VIC, 3086, Australia. ^6^Research Centre for Infectious Diseases, Department of Molecular and Biomedical Science, University of Adelaide, Adelaide, SA 5005, Australia.*


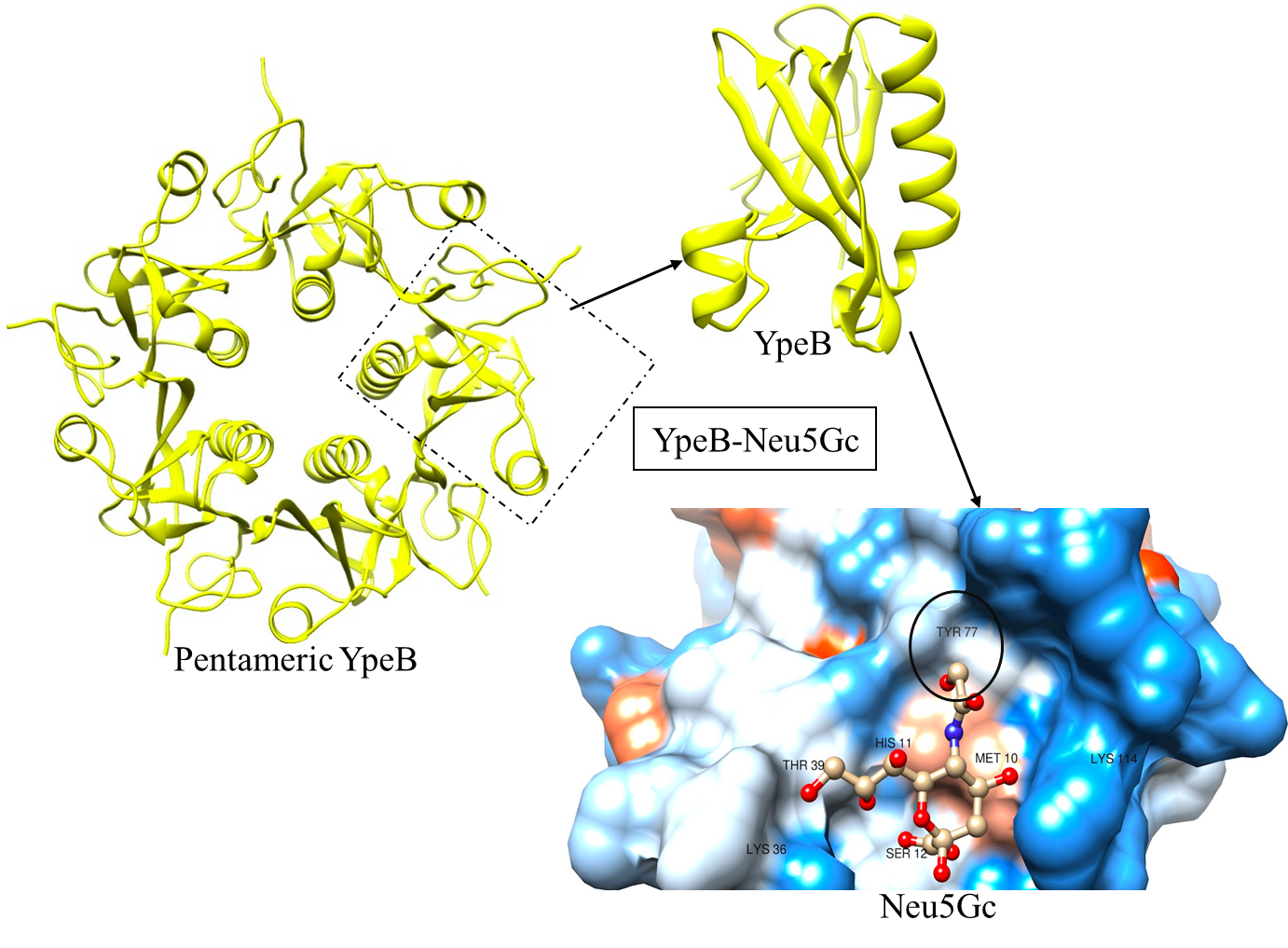


Fig. S1. Structural modelling showing interaction of Tyrosine (Tyr) residue present in sialic acid binding pocket with sialic acid binding. The circled Tyr residue at position 77 is interacting with sialic acid.


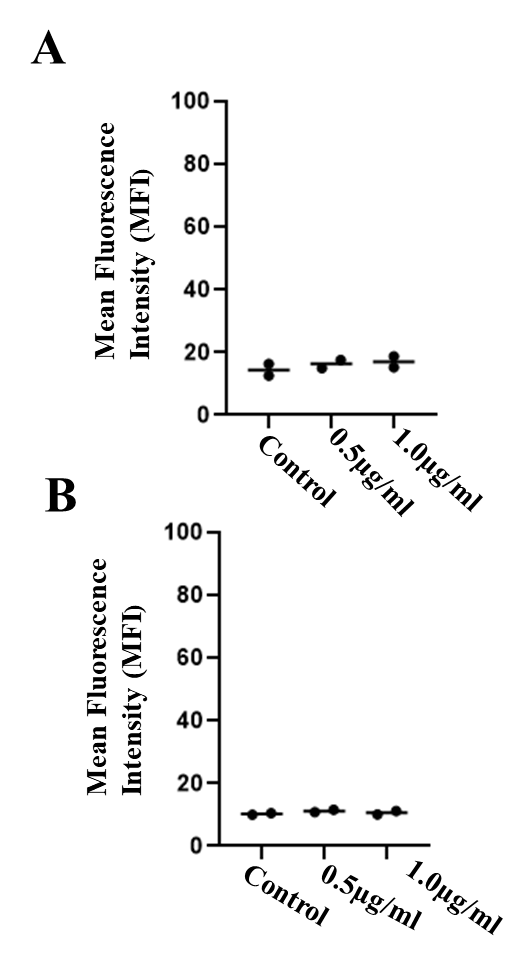


Fig. S2. Effect of YpeB mutant on cell lines. (A) U-937 cells (1x10^6^ cells) were incubated in RPMI medium with YpeB mutant (0.5 µg/ml and 1.0 µg/ml) for 16 hrs, then labeled with propidium iodide (PI) and analyzed by flow cytometry (Upper Panel). (B) COS-7 cells (1x10^6^ cells) were incubated in DMEM medium with YpeB mutant (0.5 µg/ml and 1.0 µg/ml) for 16 hrs, then labeled with propidium iodide (PI) and analyzed by flow cytometry (lower panel). The data are represented as MFI (Mean Fluorescence Intensity) for each cell line. (Please also refer to Figure 7A in the main text).


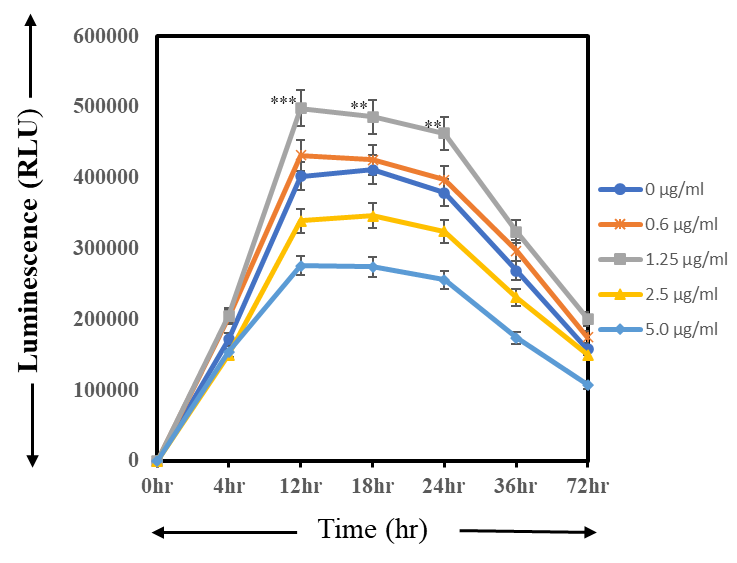


Fig. S3. Cell growth and viability of CHOK1 cells (5000 cells/well) with MAL-1 lectin. CHO-K1 cells were incubated with MAL-1 concentrations ranging from 0.6–5.0 µg/ml at different time points ranging from 0–72 hrs using an MTT cell viability assay on an ELISA plate reader at 570 nm (described in experimental procedures). ***P<0.001, ** P<0.01, *P<0.05

Fig.S4. Sequences of YpeB toxin B subunit and YpeB mutant toxin B subunit.

**YpeB toxin B subunit**:

MRYLLSLSVFLIVSLNPAFAEWTGDNVEGMHSGMIINKFHSGQVDGKPYFCIEAFKPSTTITACSVKDTSIWGASYNTLYDQAMYYYTTGKRIRVYYAPDVWTNNSFVRALTANALVGFSTCISESSCFGPDRKKHKFTVHHHHHH

**YpeB mutant toxin B subunit**:

MRYLLSLSVFLIVSLNPAFAEWTGDNVEGMHSGMIINKFHSGQVDGKPYFCIEAFKPSTTITACSVKDTSIWGASYNTLYDQAMYYYTTGKRIRVYFAPDVWTNNSFVRALTANALVGFSTCISESSCFGPDRKKHKFTVHHHHHH
